# RAS in Pregnancy and Preeclampsia and Eclampsia

**DOI:** 10.1155/2012/739274

**Published:** 2012-12-30

**Authors:** M. Rodriguez, J. Moreno, J. Hasbun

**Affiliations:** ^1^Department of Obstetrics and Gynecology, Universidad de Valparaiso, 2341131 Valparaiso, Chile; ^2^Fetal Medicine Unit, Hospital Carlos Van Buren, 2341131 Valparaiso, Chile; ^3^Fetal Medicine Unit, Hospital Clinico Universidad de Chile, 8380456 Santiago, Chile

## Abstract

Preeclampsia is a common disease of pregnancy characterized by the presence of hypertension and commitment of many organs, including the brain, secondary to generalized endothelial dysfunction. Its etiology is not known precisely, but it involved several factors, highlighting the renin angiotensin system (RAS), which would have an important role in the origin of multisystem involvement. This paper reviews the evidence supporting the involvement of RAS in triggering the disease, in addition to the components of this system that would be involved and how it eventually produces brain engagement.

## 1. Introduction

Preeclampsia is a major complication of pregnancy and corresponds to a major cause of both maternal and fetal morbidity and mortality [1–3]. It is a condition that produces a compromise of many organs, including the brain causing seizures, a condition known as eclampsia [4, 5]. The pathophysiology is not well understood, but it involves different factors, such as genetic, immunological, and inflammatory [6, 7]. In recent years there is a series of studies linking the renin angiotensin system (RAS) with preeclampsia [8–10], in the sense that the alteration of this system would be involved in the pathogenesis of this disease, as this could trigger the different characteristics in this pathology, including brain involvement.

## 2. RAS in Normal Pregnancy

RAS is a system that functions as an important regulator of blood pressure, electrolyte balance, and fluid homeostasis [11]. This system comprises the inactive peptide angiotensinogen, which is converted to angiotensin I and then the active peptide angiotensin II (Ang II) through the action of renin and angiotensin-converting enzyme (ACE) [12]. Ang II exerts its action primarily through the AT1 receptor, located widely in different tissues, including the syncytiotrophoblast [10].

During pregnancy usually occurs overexpression of many components of the RAS, both in the blood and tissues. There is an increase in plasma renin mainly by extrarenal production [13]. There is also a higher-level production of angiotensinogen liver secondary to increased circulating estrogens. ACE is the only component that has been shown to decrease during normal pregnancy, but equally there is a higher plasma concentration of Ang II [8, 13].

There is an upregulation of RAS components during normal pregnancy, but there is also a decrease in sensitivity to Ang II, whereby these women are resistant to the pressor effect of this molecule, requiring twice Ang II by intravenous infusion compared with nonpregnant women to achieve a similarly vasomotor response [14].

It is thought that this might be related to the monomer structure of AT1 during uncomplicated pregnancies, unlike the heterodimeric structure observed in terms of sensitivity to Ang II [15]. In addition, estrogens produce a shift in the formation of angiotensin peptides, reducing the formation of Ang II and increasing the production of Ang-(1–7), which has a vasodilator role [16].

Furthermore, in addition to a systemic RAS, RAS also exists in uteroplacental territory [13]. This unit consists of a placental portion, corresponding to fetal tissue, and a decidual, which is of maternal origin, and in both all components of the RAS are secreted. Therefore, there are 2 RAS systems: placental and decidual. The latter could be related to the pregnancy-associated vascular remodeling of the spiral arteries [17].

## 3. Pathophysiology of Preeclampsia: Role of RAS

Preeclampsia corresponds to a multisystem disorder characterized by increased peripheral vascular resistance, increased platelet aggregation, and systemic endothelial dysfunction [18]. Corresponds to a multifactorial disease, involving genetic and environmental components, a defective extravillous trophoblast invasion, an impaired immune tolerance between maternal, fetal and placental tissues and maternal inflammatory disorders [19, 20]. Clinically it is characterized by the presence of hypertension and proteinuria from the 2nd half of pregnancy, and the only effective treatment is the termination of pregnancy [21].

From a physiological point of view, preeclampsia is defined as a disease of two stages [22]. The first is the placental stage that occurs during the first 20 weeks of gestation. In this, the phenomena of remodeling of the vascular walls of the spiral arteries do not develop properly, resulting in abnormal placentation, thus prompting ischemic placenta [23]. The second stage occurs during the second half of pregnancy and is known as the systemic stage. This is the clinical stage of preeclampsia, in which there is an exaggerated maternal systemic inflammatory response and endothelial dysfunction as a central element [24–26]. Between these two stages are some mediators, which are understood as molecules released by the placenta and are capable of transmitting this placental damage and translate into a systemic involvement. Mediators most studied are oxidative stress, microfragments of syncytiotrophoblast (STBM), and antiangiogenic proteins [27].

There is a considerable amount of evidence supporting the role of angiogenic factors in triggering preeclampsia, and these are the tyrosine-like soluble factor (sFlt-1) and soluble endoglin (s-Eng) [28, 29]. These molecules bind to angiogenic proteins such as VEGF and prevent them from joining their membrane receptors on endothelial cells, leading to endothelial dysfunction [30]. It was observed that these factors are elevated about 6–8 weeks before the start of the clinical picture of preeclampsia, and their plasma concentrations are related to the severity of the disease [31, 32]. In animal models it has been found that inoculation of these can produce hypertension, proteinuria, and hepatic involvement, symptoms characteristic of preeclampsia [33, 34]. It is also observed that hypoxia causes increased secretion of these factors [35].

In patients with preeclampsia, dysregulation has been observed in the RAS compared to healthy pregnancies. The levels of renin, Ang I, and Ang II are lower than in uncomplicated pregnancies [36]. Despite this decrease in the expression of RAS components, in patients suffering from preeclampsia increased sensitivity to Ang II exists, showing an exaggerated pressor response to Ang II.

## 4. AT1 Receptors Autoantibodies in Preeclampsia

In recent years there is a wealth of evidence supporting the AT1 autoantibodies (AT1-AA) in the pathogenesis of preeclampsia. These correspond to IgG autoantibodies that bind to a seven-amino acid sequence present on the second extracellular loop of the AT1 receptor [37, 38]. They are present in the plasma of patients with preeclampsia and are able to increase the beating rate of the cultured cardiomyocytes [39]. Many research papers show that these autoantibodies are elevated in patients with preeclampsia, but not in uncomplicated pregnancies.

*In vitro* and *in vivo* studies have determined its role in triggering preeclampsia. These antibodies bind to the AT1 receptors of different cell groups, triggering its pathological action [40]. It has been observed that in human trophoblast cells AT1-AA substances induce the generation of reactive oxygen species (ROS) intracellularly through NDPH oxidase activation [41]. In addition, these same cells stimulate the release of PAI-1, resulting in a decreased trophoblast invasiveness [42, 43], generating a defect of placentation. This increase in PAI-1 is also observed in mesangial cells, which can produce a decrease in extracellular matrix degradation and increased subendothelial fibrin deposition thereby determining renal damage leading to proteinuria and a decreased glomerular filtration rate [44]. It has also been observed that AT1-AA binds to endothelial and vascular cells, causing endothelial damage and vasoconstriction [45, 46].

All these actions could explain endothelial dysfunction, increased peripheral vascular resistance, and impaired coagulation system observed in preeclampsia.

In animal models it has been observed that the inoculation of AT1-AA from patients with preeclampsia is capable of reproducing the characteristics of the disease [47, 48]. Reports in rats showed that surgically induced placental ischemia may cause increased levels of AT1-AA and trigger hypertension and proteinuria [49–51]. It was also observed that these autoantibodies stimulate the release of sFlt-1 and s-eng by the placenta, key proteins in triggering endothelial dysfunction [52, 53].

In the same way pregnant human studies show that placental perfusion abnormality, evaluated with Doppler ultrasound of the uterine arteries, is associated with increased plasma concentrations of AT1-AA before the onset of the disease and that plasmatic levels of these autoantibodies correlate with the severity [54]. The concentration of these autoantibodies is higher in cases of severe preeclampsia, also having a linear correlation with proteinuria and hypertension. This is further confirmed by the fact that in milder cases, as moderate preeclampsia and gestational hypertension, levels of AT1-AA are higher than in normotensive pregnancies, but lower than in severe preeclampsia. Therefore, AT1-AA would be a key element in the pathogenesis of preeclampsia and modulate the secretion of important factors responsible for the pathophysiology [55].

## 5. Eclampsia: Loss of Autoregulation of Cerebral Blood Flow and Possible Role of RAS

One of the most serious complications of preeclampsia is eclampsia, which corresponds to the presence of seizures in the context of a patient with hypertension and proteinuria [5, 56]. However, currently the eclampsia is being seen as a manifestation of a much larger entity than pregnancy, known as posterior reversible encephalopathy (PRES), which is produced by other conditions such as hypertensive encephalopathy or use of immunosuppressive drugs. In these cases there is a characteristic increase in blood pressure and/or alteration of endothelial permeability [57–60].

The PRES is characterized by the presence of well-defined signs and symptoms associated with the presence of specific neuroimaging. Among the symptoms are headache, nausea, vomiting, visual disturbances, and seizures [61, 62]. Diagnosis is by observation of symmetrical hyperintense lesions and bilateral parietooccipital level on MRI, suggestive of vasogenic edema [63].

The exact pathophysiology of PRES is not known with certainty, but is thought to be due to alterations of the vasculature and cerebral perfusion [64]. With the increase in blood pressure, brain responds with its vasculature vasoconstriction, which determines an increase of cerebral perfusion pressure (CPP). As this CPP remains persistently high, it will produce a pressure transmission to the distal small cerebral vessels, causing endothelial damage and muscle dysfunction of cerebral vascular territory [65, 66]. This phenomenon is known as barotrauma, corresponding to forced dilation of arterioles and distal opening of endothelial tight junctions, determining a disruption of the blood-brain barrier (BBB), which leads to increased permeability of this, resulting in a vasogenic edema [67].

However, not all cases of PRES relate to alterations in arterial pressure. One study shows that 16% of cases of eclampsia occur in normotensive patients, and only 13% of cases were associated with severe hypertension [68, 69]. Therefore, the loss of autoregulation of cerebral perfusion secondary to hypertension does not explain all cases of PRES, so that alteration of endothelial permeability by disruption of the BBB ought to play an important role [67]. A recent study shows that preeclampsia altered BBB permeability independently of blood pressure. In this report it is determined that plasma from patients with preeclampsia significantly increases the permeability of the BBB, compared to plasma from patients with normal pregnancies [70]. These findings support the concept of the pathophysiology of PRES that is determined by hemodynamic factors and factors altering endothelial function.

Various reports have identified the involvement of RAS in the blood-brain barrier disruption in other medical conditions [71]. Therefore, the presence of AT1-AA in patients with preeclampsia could play a key role in triggering PRES. It is shown that these autoantibodies produced an increase in peripheral vascular resistance and hypertension, which leads to a systemic endothelial dysfunction via ROS secretion and antiangiogenic proteins [37, 40], so directly involved in the conditions that can trigger PRES.

## 6. Management of Preeclampsia and Eclampsia Prevention

Currently, the only effective treatment for preeclampsia is the termination of pregnancy, which in very early pregnancies may not be the best alternative, as this results in increased perinatal morbidity and mortality secondary to prematurity [72]. Therefore, it is important to seek management strategies from the physiological point of view, and in that sense, the management of RAS alteration appears to be a logical choice, given the strong evidence about an excessive AT1 receptor activation during preeclampsia.

Regarding eclampsia, the drug of choice for prevention and management is magnesium sulfate. This drug reduces the risk of seizures in patients with severe preeclampsia [73]. Its mechanism of action is not entirely clear, but it has been shown to be capable of reducing the CPP [74]. However, it should be administered intravenously and usually for 48 hrs, in patient hospitalized and only for short periods of time.

Many studies determined that the addition of losartan (AT1 receptors blocker) or neutralizing antibodies of the AT1-AA (7-amino acid peptide epitope or 7-aa) blocks the effect of AT1-AA, further confirming that the action of these antibodies is through activation of AT1 receptors (z). Animal studies with surgically induced placental ischemia demonstrate that the addition of these compounds significantly reduces arterial pressure [49], but this effect was not seen with ACE inhibitors [75]. In another report, AT1-AA purified from pregnant women was injected in mice. They triggered hypertension and proteinuria, were significantly reduced or abolished when administered losartan or 7-aa [47]. Adoptive transfer studies in pregnant mice have demonstrated that release of antiangiogenic factors and proinflammatory cytokines resulting from autoantibody-mediated receptor activation is blocked by the addition of 7-aa or AT1 receptor antagonist [10, 37].

The main problem with AT1 receptors blockers is that they increase the risk of fetal malformations, such as oligohydramnios, pulmonary hypoplasia, transient renal failure, preterm delivery, and Potter syndrome [76–78], so its use during pregnancy should be avoided. However, AT1 receptors blockers can be used postpartum, and considering that currently about 30% of eclampsia occur in postpartum [56, 79] and that AT1-AA levels can remain high for a long time [80], AT1 receptors blockers become an interesting strategy for the management of hypertension during the postpartum period and to decrease the incidence of eclampsia, as well as controlling blood pressure; they block the action of endothelial AT1-AA, which is still present in the postpartum period. 

Neutralizing antibodies 7-aa have the advantage of not inhibiting the AT1 receptor; they only block the AT1-AA, without changing completely the action of Ang II. Therefore, it seems a useful strategy for the management of preeclampsia. However, we must await the development of further studies to determine its safety during pregnancy.

## 7. Discussion

During preeclampsia there is an alteration of the RAS. The presence of AT1-AA determines triggering a series of actions in tissues and organs that would result in an increase in peripheral vascular resistance, altered coagulation, renal impairment, and systemic endothelial dysfunction. These alterations can generate the commitment of many organs, including the brain, producing a PRES.

Blocking the action of these autoantibodies using losartan or 7-aa substantially decreases the damage caused by AT1-AA, creating an opportunity for the management and prevention of complications of preeclampsia.
